# Phylogeography of *Liquidambar styraciflua* (Altingiaceae) in Mesoamerica: survivors of a Neogene widespread temperate forest (or cloud forest) in North America?

**DOI:** 10.1002/ece3.938

**Published:** 2014-01-10

**Authors:** Eduardo Ruiz-Sanchez, Juan Francisco Ornelas

**Affiliations:** 1 Departamento de Biología Evolutiva, Instituto de Ecología, A.C.Xalapa, Veracruz, 91070, México; 2 Red de Biodiversidad y Sistemática, Centro Regional del Bajío, Instituto de Ecología, A.C.Pátzcuaro, Michoacán, 61600, México

**Keywords:** Altingiaceae, cloud forests, *Liquidambar*, Mesoamerica, Mexico, Pleistocene, Trans-Mexican Volcanic Belt

## Abstract

We investigate the genetic variation between populations of the American sweetgum (*Liquidambar styraciflua*), a tree species with a disjunct distribution between northeastern Texas and Mexico, by analyzing sequences of two chloroplast DNA plastid regions in Mesoamerica. Our results revealed phylogeographical structure, with private haplotypes distributed in unique environmental space at either side of the Trans-Mexican Volcanic Belt, and a split in the absence of gene flow dating back ca. 4.2–1.4 million years ago (MYA). Species distribution modeling results fit a model of refugia along the Gulf and Atlantic coasts but the present ranges of US and Mesoamerican populations persisted disjunct during glacial/interglacial cycles. Divergence between the US and Mesoamerican (ca. 8.4–2.8 MYA) populations of *L. styraciflua* and asymmetrical gene flow patterns support the hypothesis of a long-distance dispersal during the Pliocene, with fragmentation since the most recent glacial advance (120,000 years BP) according to coalescent simulations and high effective migration rates from Mesoamerica to the USA and close to zero in the opposite direction. Our findings implicate the Trans-Mexican Volcanic Belt as a porous barrier driving genetic divergence of *L. styraciflua*, corresponding with environmental niche differences, during the Pliocene to Quaternary volcanic arc episode 3.6 MYA, and a Mesoamerican origin of populations in the USA.

## Introduction

Mesoamerican cloud forests encompass an extremely diverse and heterogeneous mixture of temperate and tropical species. Several of the temperate species (ca. 50 plant lineages) exhibit a disjunction between the temperate floras of the eastern USA and the mountains of eastern Mexico (Miranda and Sharp 1950; Graham 1973, 1999). Two models can explain the origin of disjunct distribution for Mexican species that have a north temperate affinity: (1) vicariance due to range contraction of a one widespread temperate forest (or cloud forest) in North America, in which the Mexican temperate element represents “survivors” since the early or middle Eocene (Axelrod 1975) and/or to fragmentation of the temperate forest after repeated glaciations in the Quaternary (Zhao et al. 2013), or (2) long-distance dispersal between suitable habitats, in which Pleistocene cooling forced temperate elements in the southeastern USA further south into peninsular Florida and the mountains of eastern Mexico (Deevey 1949; Morris et al. 2008, 2010), a north-to-south introduction in the Neogene due to temperature decline (Graham 1973, 1999), or a late Paleogene-to-early Neogene colonization time of these taxa in Mexico that later isolated by more arid conditions during the Pliocene (Braun 1950). Graham (1999) noted that the appearance of these temperate woody taxa is consistent with a major temperature decline in the mid-Miocene before which very few of these elements are found in the northern Mexican palynofloras (see also Hoey and Parks 1994). He suggested that the absence of temperate pollen from earlier Mexican records and the presence of temperate pollen in the Eocene record of the southeastern USA provide evidence for a north-to-south introduction for the Mexican temperate element. Fossil pollen data support Braun's hypothesis of early arrival, indicating that the occurrence of temperate woody genera in the Mexican palynofloras as early as the mid-Pliocene (Graham 1973, 1999). Then, if the arrivals of temperate taxa into eastern Mexico were the result of range expansion from the southeastern USA in response to climate cooling, the observed modern disjunction would be a consequence of repeated expansions and contractions in response to Pleistocene glacial/interglacial cycles.

**Figure 1:**
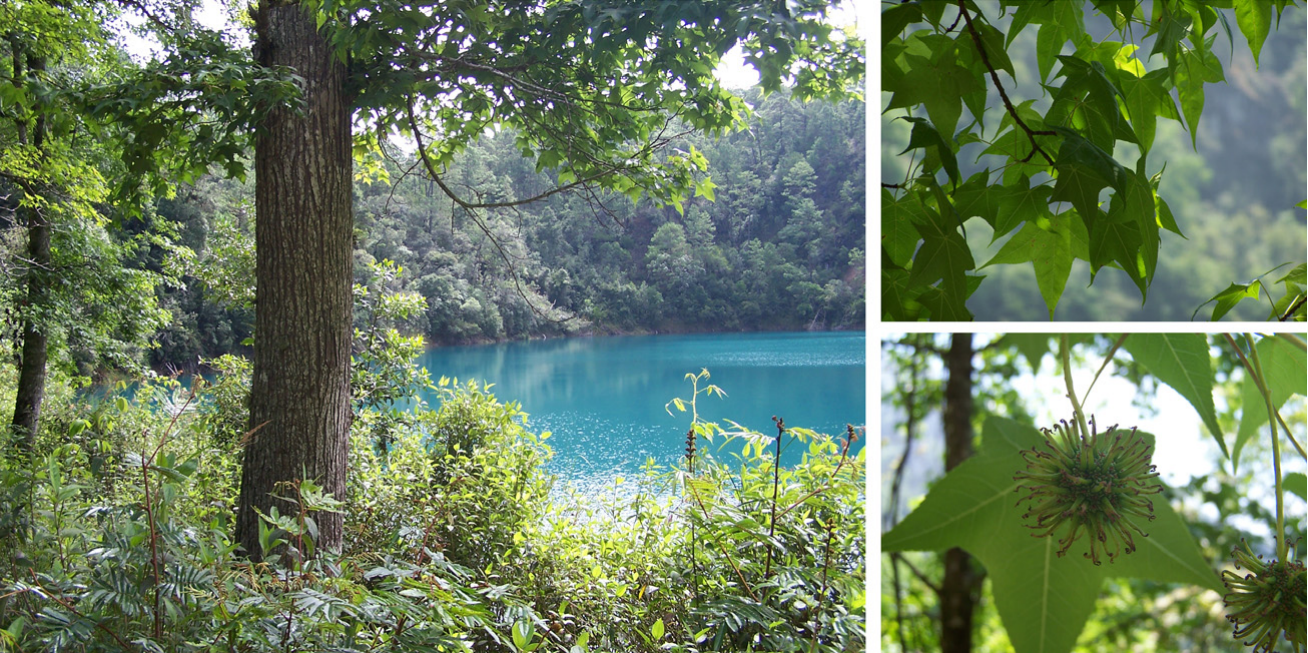
American sweetgum (*Liquidambar styraciflua*) at Lagunas de Montebello, Chiapas, (left), and Tlanchinol, Hidalgo, Mexico (right). Photographs by Eduardo Ruiz-Sanchez.

*Liquidambar styraciflua* L. [Altingiaceae] is a common tree with a disjunct distribution between the deciduous forests of the southeastern USA and the Mesoamerican cloud forests (Fig. 1; Sosa 1978; Morris et al. 2008). The relationship between *Liquidambar* populations from the USA and Mexico is currently under debate (Hoey and Parks 1994; Ickert-Bond and Wen 2006; Morris et al. 2008). Morris et al. (2008) used cpDNA sequences of 117 *L. styraciflua* individuals from 22 US populations and recovered two intraspecific clades, one potentially originating along the US Gulf Coast and the other from the highlands of the Cumberland Plateau and the Southern Appalachians, which diverged ca. 8 MYA. Only two haplotypes were recovered from two additional Mesoamerican populations, one was unique to one population and the other shared by both populations was one of the most common haplotypes recovered within and between US populations. Here, we used a broader geographic sampling of Mesoamerican populations (160 individuals from 19 populations in Mexico) to the cpDNA dataset in the study by Morris et al. (2008). Using both cpDNA sequence data, and fossil-calibrated genealogies, coalescent-based divergence time inference and estimates of gene flow, we attempt to distinguish between two phylogeographical scenarios: a Pleistocene refugial pattern in the southeastern USA, as described for several taxa (Soltis et al. 2006; Morris et al. 2010), in which the disjunction between the US and Mexican populations of *L. styraciflua* is younger than the genetic signal observed by Morris et al. (2008), or a Miocene arrival of the temperate element into Mexico (Braun 1950; Graham 1999) consistent with fossil data in which Mexican populations are relatively old, while the disjunction between the US and Mexican populations should be relatively recent. Because some phylogeographical breaks might have originated much later, we also used ecological niche models to predict Pleistocene distributions based on palaeoclimatic data reconstructions and tests of niche conservatism to assess the geography of dispersal. A palaeodistribution pattern would indicate that both the US and Mesoamerican populations persisted disjunct in their present ranges through the later Pleistocene rather than a pattern of expansion to the south.

## Materials and Methods

### Study system

*Liquidambar styraciflua* is a deciduous timber tree currently used for reforestation, agroforestry, and landscaping (e.g., Pedraza and Williams-Linera 2003), and the tree sap for medicinal and incense production (Peterson and Peterson 1992). It is a relatively fast-growing pioneer species, has an average lifespan of 200 years, and reaches reproductive maturity at 20–30 years of age (Morris et al. 2008). This monoecious tree has wind-pollinated flower production in mid-to-late spring and fruit production in late fall (Sosa 1978). Fruits open to release wind-dispersed seeds, which are also eaten by birds, squirrels, and chipmunks (Morris et al. 2008). Some authors recognize morphological variants similar to *L. styraciflua* occurring in Mexico to Honduras as *L. macrophylla* Oerst. and *L. styraciflua* var. *mexicana* Oerst. (Sosa 1978; Zhang et al. 2003). Using cpDNA sequences from five noncoding regions, Ickert-Bond and Wen (2006) showed that *L. macrophylla* from Mesoamerica formed a well-supported clade with *L. styraciflua* from eastern North America. Morphologically, *L. macrophylla* has slightly larger leaves and fruits, and occurs in the cloud forests at high elevations (ca. 370–2300 m) in Mexico, as compared to *L. styraciflua* from eastern North America, which ranges from sea level to 300 m in elevation (Ickert-Bond and Wen 2006). However, most researchers recognize only one species that exhibits considerable variation in leaf and infructescence size (e.g., Morris et al. 2008).

### Samples and DNA sequencing

**Figure 2:**
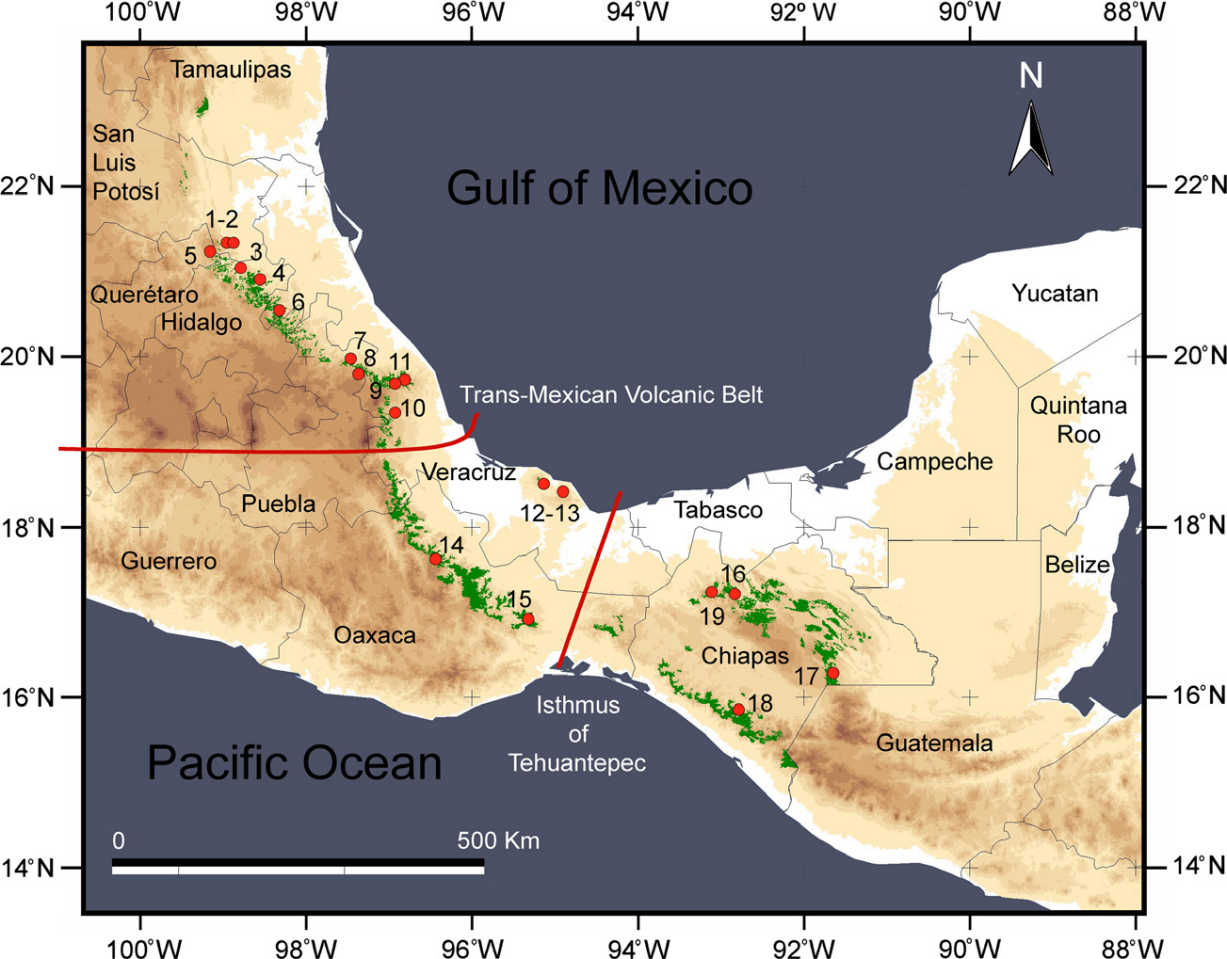
Geographic distribution of cloud forest in eastern and southern Mexico, which corresponds to the distribution of *Liquidambar styraciflua*. Small red circles with numbers represent the collection sites. Refer to Table S1 for cloud forest areas and locality codes.

Leaf tissue samples were collected from 160 *L. styraciflua* individuals in 19 populations throughout the species range in cloud forests of Mexico, from 378 to 2100 m above sea level (Fig. 2, Table S1). Thirteen populations were sampled from three disjunct cloud forests areas along the Sierra Madre Oriental, two in Los Tuxtlas region (Sierra de Los Tuxtlas and Sierra de Santa Marta, TUX), and four populations from Chiapas highlands (Fig. 2). Most populations collected have an accompanying herbarium voucher that is deposited at the Instituto de Ecología, A.C. herbarium (XAL). Leaf material was obtained from 2 to 14 individuals at each locality, and leaf tissue samples were preserved in silica gel desiccant (Chase and Hills 1991) until DNA extractions were performed.

Two chloroplast DNA plastid regions with the highest base pairs—327 base pairs (bp) of *psb*A*–trn*H and 1090 bp of *psb*E–*pet*L—were used because they proved to exhibit the most variation in Morris et al. (2008) and because consistent polymerase chain reaction (PCR) amplification. Screening of the nuclear ribosomal internal transcribed spacer spacer (*ITS*) region revealed almost no sequence variation across 18 samples from geographically distant locations of *L. styraciflua* in Mexico (E. Ruiz-Sanchez, unpubl. data). PCR and sequencing protocols of the chloroplast DNA plastid regions can be found in Data S1. Forward and reverse sequences were assembled using Sequencher 4.9 (Gene Codes, Ann Arbor, MI, USA) and subsequently aligned with SE-AL ver. 2.0a11 (http://tree.bio.ed.ac.uk/software/seal/). The sequences reported in this study are available from GenBank (accession nos KF009884–KF009912, KF009913–KF009941). We additionally obtained GenBank sequences of *L. styraciflua* from Morris et al. (2008) to be used in the study (EF138707–EF138728, EF138683–EF138704).

### Relationships among haplotypes

A statistical parsimony network of haplotypes was constructed using TCS ver. 1.2.1 (Clement et al. 2000) with a 95% connection limit and treating gaps as missing data for the US–Mesoamerica dataset and as fifth character state for the Mesoamerica dataset. Loops were resolved according to the topology, frequency, and geographic criteria proposed by Pfenninger and Posada (2002).

We also used Bayesian inference (BI) analyses on the combined dataset to infer evolutionary relationships among haplotypes using MRBAYES ver. 3.1.2 (Huelsenbeck and Ronquist 2001). jMODELTEST ver. 0.1.1 (Posada 2008) was run to choose the model of molecular evolution that best fitted our combined sequence data under the Akaike information criterion (AIC), TVM+I (proportion of invariable sites = 0.6470). Details of the BI analysis and outgroup justification can be found in Data S1.

### Estimation of divergence times

To relate genetic differentiation found among *L. styraciflua* haplotypes to pre-Pleistocene and Pleistocene events, we estimated divergence time under a Bayesian approach as implemented in BEAST ver. 1.6.1 (Drummond and Rambaut 2007). We estimated the divergence time to the Altingiaceae using the *psb*A*–trn*H and *psb*E–*pet*L sequences of Morris et al. (2008) plus *L. styraciflua* haplotypes from Mexican populations. We used the model of sequence evolution GTR+I based on the results of AIC model selection from jMODELTEST for this analysis. Given the predominantly intraspecific nature of our data, an uncorrelated lognormal relaxed clock model and a coalescent model assuming constant size were used to model the tree prior. *Microaltingia* from the Late Cretaceous of New Jersey (Zhou et al. 2001) was used to calibrate the root node, approximating a median age of 90 MYA (lognormal distribution, mean 0.0, SD 1.0, offset 90, range 97–90 MYA). Based on the western North American Middle Miocene *Liquidambar changii* (Pigg et al. 2004), we constrained the divergence of the clade *L. acalycina* and *L. formosana* + *A. obovata* from the rest of the eastern Asian clade to be minimally 15.6 MYA (lognormal distribution, mean 0.0, SD 1.5, offset 15.6, range 34.5–15.6 MYA). For the *L. styraciflua* crown group, we set an age of 3 MYA on the basis of fossil material (Morris et al. 2008) from the Citronelle formation of southern Alabama (lognormal distribution, mean 0.0, SD 1.0, offset 3, range 10.1–3.1 MYA). For divergence time estimations, the Markov chain Monte Carlo (MCMC) was run for three independent 10 million generations, sampling every 1,000 generations. The output was visualized using TRACER ver. 1.5 (http://tree.bio.ed.ac.uk/software/tracer/), making sure that parameter values were fluctuating at stable levels. Based on these results, the first 3000 trees were discarded as burn-in, and the remaining samples were summarized as a maximum clade credibility tree with mean divergence times and 95% highest posterior density (HPD) intervals of age estimates in TREEANNOTATOR. Finally, these results were summarized in a single tree visualized in FIGTREE ver. 1.3.1 (http://tree.bio.ed.ac.uk/software/figtree/).

### Population indices and geographic structure of populations

We calculated nucleotide diversity (*π*) and haplotype diversity (*h*) for the resulting groups, and computed pairwise comparisons of *F*_ST_ values between populations and groups in ARLEQUIN ver. 3.01 (Excoffier et al. 2005) with 1000 permutations. Population diversity for unordered (*h*_S_, *h*_T_) and ordered haplotypes (*v*_S_, *v*_T_) and differentiation parameters (*G*_ST_, *N*_ST_) were estimated according to the methods described by Pons and Petit (1996) using PERMUT ver. 1.0 (Pons and Petit 1996).

Analysis of molecular variance (AMOVA; Excoffier et al. 1992) based on pairwise differences (Tamura and Nei) from the concatenated cpDNA dataset was performed to assess genetic differentiation between locations (a) grouped into USA and Mesoamerica, and for the Mesoamerican samples with locations (b) treated as a single group to assess genetic differentiation within and among locations, (c) grouped into north and south of the Trans-Mexican Volcanic Belt (TMVB; see Results), and (d) grouped into three groups (north and south of the TMVB and TUX; Fig. 2 and Table S1) using ARLEQUIN. Significance tests were conducted using 10,000 permutations.

We also conducted a spatial analysis of molecular variance (SAMOVA) implemented in SAMOVA ver. 1.0 (Dupanloup et al. 2002) as a means to infer possible other groups without user-defined structure parameters. SAMOVA attempts to reconstruct groups of locations that are geographically homogeneous and genetically differentiated from each other, maximizing the proportion of total genetic variance due to differences between groups of locations (*Φ*_CT_). The most likely number of groups (*K*) was determined by repeatedly running the software SAMOVA with two-to-five groups and choosing those partitions with a maximum *Φ*_CT_ value, as suggested by Dupanloup et al. (2002). We explored *K*-values with one hundred simulated annealing simulations for each *K*.

### Historical demography

Three methods were used with the concatenated cpDNA dataset to infer the historical population expansion of *L. styraciflua* in Mesoamerica. First, Tajima's *D* (Tajima 1989) and Fu's *F*_*s*_ (Fu 1997) neutrality tests were examined to detect departures from a constant population size under the neutral model using ARLEQUIN with 10,000 permutations. In the neutrality tests, population growth was indicated by significant negative values. Second, mismatch distributions (Rogers and Harpending 1992) calculated in DNASP ver. 5 (Rozas et al. 2003) with the sudden demographic/population expansion model (Rogers 1995) with 10,000 permutations. Statistically significant differences between observed and simulated expected distributions were evaluated with the sum of the square deviations (SSD). Third, Bayesian skyline plots (BSP; Drummond et al. 2005) were performed in BEAST ver. 1.5.4 (Drummond and Rambaut 2007; Heled and Drummond 2010) to describe demographic history by assessing the time variation in effective population size. Three independent runs of 10 million generations were performed using the substitution model F81 (HKY) with empirical base frequencies, an uncorrelated lognormal relaxed clock model, and a piecewise-constant coalescent Bayesian skyline tree prior with five starting groups. Trees and parameters were sampled every 1,000 iterations, with a burn-in of 10%. The time axis was scaled using the rates of 1.0–3.0 × 10^−9^ substitutions per site per year (s/s/y) for chloroplast-wide, synonymous substitution rates described for most angiosperms (Wolfe et al. 1987) and the rate of 1.59 × 10^−9^ s/s/y calculated for the cpDNA loci in *Liquidambar* (see below). The results of each run were visualized using TRACER (http://tree.bio.ed.ac.uk/software/tracer/) to ensure that stationarity and convergence had been reached (ESS > 200). BSPs were generated for the groups north and south of the TMVB.

We used the computer program IMa (Hey and Nielsen 2007) on *L. styraciflua* genetic groups (US versus Mesoamerica, north versus south of the TMVB) to estimate the time of divergence (*t*) between populations, effective number of migrants per generation (*m*_1_ and *m*_2_), and the effective population size of the ancestral (*q*_A_) and descendant populations (*q*_1_ and *q*_2_). The isolation-with-migration (IM) model (Nielsen and Wakeley 2001; Hey and Nielsen 2004), which assumes that an ancestral population was divided into two descendant populations with the possibility that gene interchange occurs between the two new populations, is appropriate to estimate parameters for two populations that have diverged recently and that may be sharing haplotypes as a result of gene interchange. We began with multiple runs of 10,000 steps (following 100,000 iterations as burn-in) to assess mixing and to fine-tune the parameter space. We then conducted the simulation for a burn-in of one million generations and 30 million steps, under the HKY model of sequence evolution. Three independent runs were performed with different seed numbers to guarantee convergence of samples (Hey and Nielsen 2004). We considered that the analyses had converged upon a stationary distribution if the independent runs had similar posterior distributions (Hey 2005) and the effective sample size (ESS) for each parameter was at least 100 (Kuhner and Smith 2007). We report the parameter estimates of one run and the 90% highest posterior densities (HPD) intervals of each parameter. Because an appropriate chloroplast nucleotide substitution rate for *L. styraciflua* has not been calibrated, we used the substitution rates 1.0 × 10^−9^ and 3.0 × 10^−9^ s/s/y (Wolfe et al. 1987) to estimate the effective population sizes (Ne) of each genetic group. Using DNASP with Jukes and Cantor correction, a substitution rate specific for the cpDNA loci in this study was also calculated as the sequence divergence divided by two lineages (*D*_*xy*_ = 0.00757/2 = 0.003785 s/s/l) divided by two times the divergence time (11.9 Ma) among *Liquidambar* species (0.003785/23.8 = 0.000159/1,000,000) to compare estimation of effective population sizes with those based on general rates from Wolfe et al. (1987). The estimated value of 1.59 × 10^−9^ s/s/y lies within the mutation rate range estimated by Wolfe et al. (1987). The mutation rates were converted to per locus rate by multiplying by the fragment length in base pairs for conversion to demographic units, as required by IMa (Hey and Nielsen 2007). To convert the effective populations size estimates, we used a either 34 or 124 year generation time based on the observation that the age at maturity (seed production) begins approximately 25 years after seed germination (Morris et al. 2008; U.S. Department of Agriculture, Forest Service Intermountain Research Station's Fire Sciences Laboratory at http://willow.nefes.umn.edu accessed April 24, 2013) and an assumed high annual adult survival of 0.9 or 0.99 based on typical estimates for long-lived tree species (Franklin and Debell 1988). The approximate average generation time (*T*) is calculated according to *T* = *a *+ [*s* / (1–*s*)] (Lande et al. 2003; Spellman and Klicka 2006), where *a* is the time to maturity and *s* is the adult annual survival rate. Based on this, estimates for T range from 34 to 124 years. If we consider periodic major disturbances (e.g., hurricanes or disease outbreaks), a lower survival rate (0.9) and therefore a lower average generation time (34 years) would be more likely. We primarily assumed an average generation time of 34 years, but also explored the effects of generation times as high as 124 years. To convert the time since divergence parameter of IMa to years, *t*, we divided the time parameter (*b*) by the mutation rate per year (*u*) converted to per locus rate by multiplying by the fragment length in base pairs, and calculated for the 1.0–3.0 × 10^−9^ Wolfe's et al. (1987) estimates described for most angiosperms and the 1.59 × 10^−9^ s/s/y rate estimate using sequence divergence among *Liquidambar* species, respectively.

### Hypothesis testing of Pleistocene refugia

We used coalescent simulations to test explicit a priori hypotheses (two glacial/interglacial refugia hypothesis and the alternative single refuge-fragmentation hypothesis; Knowles 2001) concerning the Pleistocene history of *L. styracifua*. The two-refugia (or vicariance) hypothesis assumed that the US and Mesoamerica populations persisted isolated in two refugia since the Pliocene (T1 = 8,500,00 years BP; hypothesis A1; T2 = 5,200,000 years BP; hypothesis A2; see IMa results), in two Pleistocene refugia for the duration of the glacial episodes (T3 = 1,800,000 years BP; hypothesis A3), or that populations persisted in two refugia only since the most recent glacial advance (T4 = 120,000 years BP; hypothesis A4). This hypothesis predicts that population structure of *L. styraciflua* would reflect the effects of colonization from multiple refugia or long-term geographic isolation and divergence between groups. The alternative hypothesis (B1–B4) tested fragmentation of a single refugial population with the number of years in the refugium differing (B1, T1 = 8,500,000 years BP; B2, T2 = 5,200,000 years BP; B3, T3 = 1,800,000 years BP; B4, T4 = 120,000 years BP). Effective population sizes (*N*_e_) of 10,000, 50,000, 100,000, or 1,000,000 were used for all the simulations based on the values calculated with IMa, and absolute time (years) was converted to coalescent time (generations) assuming a generation time of 34 or 124 years for *L. styraciflua* using the equation from Lande et al. (2003) and parameter values as explained above.

Using MESQUITE ver. 2.75 (Maddison and Maddison 2011), hypothesis testing was performed with coalescent simulations and generating 1000 coalescent genealogies under each historical scenario. The distribution of *s*, the minimum number of sorting events required to explain the population subdivision (Slatkin and Maddison 1989), was recorded; small *s*-values indicate high concordance between the gene trees and the hypothesized population trees, whereas larger *s*-values in the observed gene tree indicate that the haplotypes are widely scattered across populations. The *s*-value of our Bayesian genealogy (not shown) was compared with the *s*-values of the simulated genealogies to evaluate model fit (see also Spellman and Klicka 2006). If the observed gene tree *s*-value falls outside the 95% confidence interval of the distribution of *s* associated with any population model, then the model can be rejected (Knowles 2001).

### Ecological niche modeling

We constructed species distribution models (SDM; Elith et al. 2011) of (1) all US populations of *L. styraciflua* (*L. styraciflua sensu stricto*), (2) only the Mesoamerican populations (*L. macrophylla sensu* Oerst.), and (3) both US and Mesoamerican populations (*L. styraciflua sensu lato*) to predict where *L. styraciflua* resided at the LGM and whether range expansion and population connectivity are observed between groups of populations. A total of 218 unique occurrence data were assembled for *L. styraciflua* using records gathered from The World Biodiversity Information Network (REMIB; http://www.conabio.gob.mx/remib_ingles/doctos/remib_ing.html; accessed June 2010) and the Global Biodiversity Information Facility (GBIF; http://www.gbif.org/; accessed September 2010), supplemented with our georeferenced records from field surveys and collection. Distributional records were input into the maximum entropy machine-learning algorithm in MAXENT ver. 3.2.2 (Phillips et al. 2006) to infer a SDM. Present climate layers (BIO 1–19) were drawn from the WorldClim bioclimatic database (Hijmans et al. 2005; http://www.worldclim.org), and MAXENT was set to randomly use 30% of values for training and 70% of values for testing the model. After removing variables that exhibited a strong correlation (Spearman's rank correlation >0.9), ten variables (see Results) were used to generate the SDM model under current climatic conditions using MAXENT with the default parameters for convergence threshold (10^−5^) and 1000 iterations. We evaluated model performance using the area under the curve (AUC) values of the threshold independent receiving operating characteristic curve (ROC). Resulting distributions were projected with QUANTUM GIS ver. 1.8.0-Lisboa. To explore distributions earlier in time, the SDM under current climatic conditions of *L. styraciflua* was projected to past climatic scenarios, at the LGM (ca. 21,000 years BP) and Last Interglacial (LIG; 140–120,000 years BP). Past climate layers were also drawn from the WorldClim webpage for two LGM past climate scenarios developed by the Paleoclimate Modeling Intercomparison Project Phase II (Braconnot et al. 2007): the Community Climate System Model (CCSM; Collins et al. 2004) and the Model for Interdisciplinary Research on Climate (MIROC; Hasumi and Emori 2004), and the LIG (Otto-Bliesner et al. 2006). Both CCSM and MIROC climate models simulate climatic conditions as they are calculated to have been for the LGM, whereby a stronger temperature decrease is assumed in CCSM compared with MIROC (Otto-Bliesner et al. 2007). Therefore, SDMs for present distribution at LGM (CCSM and MIROC) and at LIG for *L. styraciflua* are contrasted. From the dataset used above, records of Mesoamerica or records in the USA were excluded, and MAXENT was used to build the species distribution model for the present distribution at LGM and at LIG to determine whether the climatic conditions that these populations currently use today were present at the LGM and LIG. The output of MAXENT consists of grid maps with each cell having an index of suitability between 0 and 1. Low values indicate that the conditions are unsuitable for the species to occur, whereas high values indicate that the conditions are suitable.

We employed the multivariate method introduced in McCormack et al. (2010) to test for niche divergence/conservatism. Briefly, we extracted raw data from our occurrence points for the USA/Mesoamerican and north/south of the TMVB lineages and, to generate the background predictions, from 1000 random points from within the geographic ranges of each lineage. We then drew the random points from within a minimum convex polygon drawn around our occurrence sites (McCormack et al. 2010) using QUANTUM GIS ver. 1.8.0-Lisboa. Next, we conducted a PCA on these data, extracting the first three PC (niche) axes for further consideration because they comprised the bulk of the variation and were readily interpretable (see Results). Niche divergence or conservatism was evaluated on each niche axis by comparing the observed difference between the means for each lineage on that axis to the mean difference in their background environments on the same axis. A null distribution of background divergence was created by recalculating the score of background divergence over 1000 jackknife replicates with 75% of replacement. Significance for rejecting the null was evaluated at the 95% level. All analyses were conducted using Stata 10 (StataCorp 2003).

## Results

### Sequence analyses

We obtained 160 cpDNA sequences of *L. styraciflua* in Mexico. The combination of *psb*A*-trn*H and *psb*E-*pet*L produced 1417 bp with 10 variable sites (*S*). Haplotype diversity (*h*) was high for most localities ranging from 0.18 to 0.86, reflecting the presence of different haplotypes within each site (Table S2). Nucleotide diversity (*π*) was low (0.0001–0.0019) for most populations, indicating little variation between sequences from the same population (Table S2). Ten substitutions were detected, and three regions with indels of 1–3 bp were treated as fifth character state after adjusting the length of the sequences to the length of those from the US populations of the Morris et al. (2008) study for further analyses.

### Relationships among haplotypes

The statistical parsimony network of US and Mesoamerican samples recovered 24 haplotypes. The most frequent haplotype (H1) is shared by populations of *L. styraciflua* in the US and by Mesoamerican populations south of the TMVB (Mesa de la Yerba, Veracruz; Comaltepec and Chayotepec, Oaxaca; Coapilla, Jitotol, Lagunas de Montebello and Nueva Colombia, Chiapas; Guatemala; Fig. S1). Haplotype H2 is shared among individuals from populations in Chiapas (Coapilla, Jitotol), Oaxaca (Comaltepec), Veracruz (Chiconquiaco, La Cortadura), and one individual from Louisiana; H11 was exclusively found in the two populations of the TUX region; H4–H5 and H7–H10 were private to populations north of the TMVB; H3 was private to Coapilla, Chiapas; and H6 was private to population in Chayotepec, Oaxaca, both localities south of the TMVB; and H12–H24 were located exclusively in US populations (Fig. S1).

**Figure 3:**
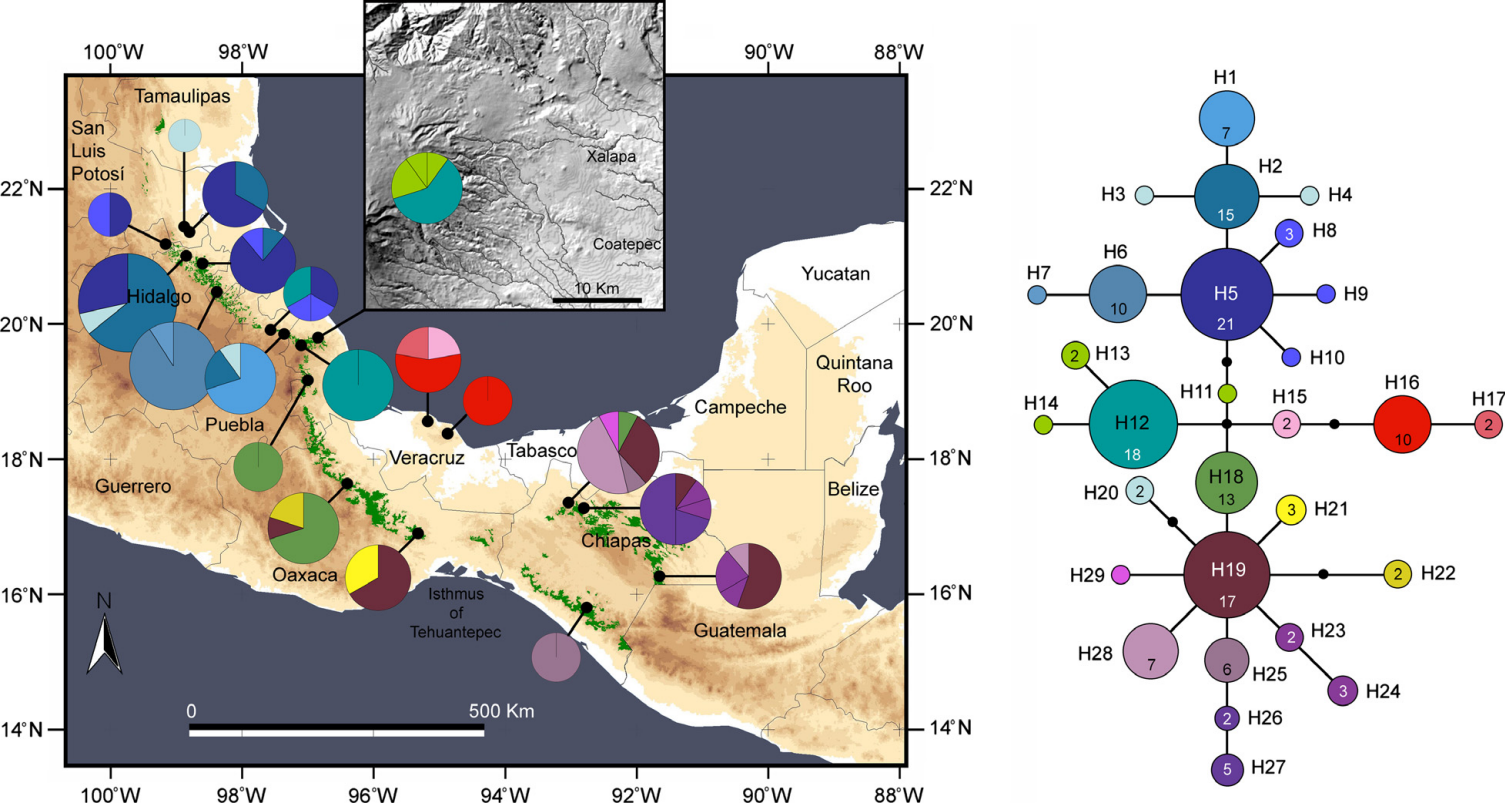
Geographic distribution of *Liquidambar styraciflua* cpDNA haplotypes in Mexico and statistical parsimony haplotype network. Current natural range of cloud forest (indicated by green shading) is overlaid on a relief map of eastern Mexico. Pie charts represent the haplotypes found in each sampling locality. The size of sections of the pie charts is proportional to the number of individuals with that haplotype. Haplotype designations in the network correspond to those in Table S1, the size of circles is proportional to the frequency of each haplotype, and small filled circles are nonsampled haplotypes. The numbers in the haplotypes indicate the number of individuals that share that haplotype. Haplotypes 18 and 19 are shared by populations in the USA. Geographic distribution and statistical parsimony haplotype network of samples from Mexico and those in the USA from Morris et al. (2008) are shown in Fig. S1.

The aligned dataset for 19 Mesoamerican populations of *L. styraciflua* yielded 29 haplotypes among 160 sequences (Table S1). Statistical parsimony retrieved a well-resolved network, in which three haplogroups can be distinguished (Fig. 3). The most frequent (13.1% of the individuals and 26.3% of the sampled populations) haplotype (H5) forms the core of the first haplogroup, from Xilitla, San Luis Potosí to Lagunillas, Puebla. H5 is connected by one or more than one step to several haplotypes (H2–H14) connecting populations north of the TMVB. Two populations in the TUX region sharing H16 formed the second group of haplotypes, with two private haplotypes to the population in the Sierra de Los Tuxtlas (H15, H17). The third group connected to H5 by more than one step consists of H19 (10.6% of the individuals and 26.3% of the sampled populations), connecting the Oaxaca populations with populations in Chiapas and Guatemala (south of the TMVB), and subgroups of haplotypes located exclusively in Oaxaca (H18, H21–22) and adjacent, but disjunct, populations from Chiapas (H23–H29).

The 50% majority consensus tree (Results not shown) grouped all *L. styraciflua* haplotypes (USA and Mesoamerica) in a single, well-supported clade (PP 0.92), supporting the monophyly of *L. styraciflua*. The basal split between clade containing haplotypes H15, H19, and H24 distributed in the Cumberland Plateau and the Southern Appalachians (T–W in Morris et al. 2008) was recovered according to Morris et al. (2008). However, no further haplotype groups were supported by the BI analysis; neither the US nor Mesoamerican populations formed separate clades.

### Estimations of divergence times

**Figure 4:**
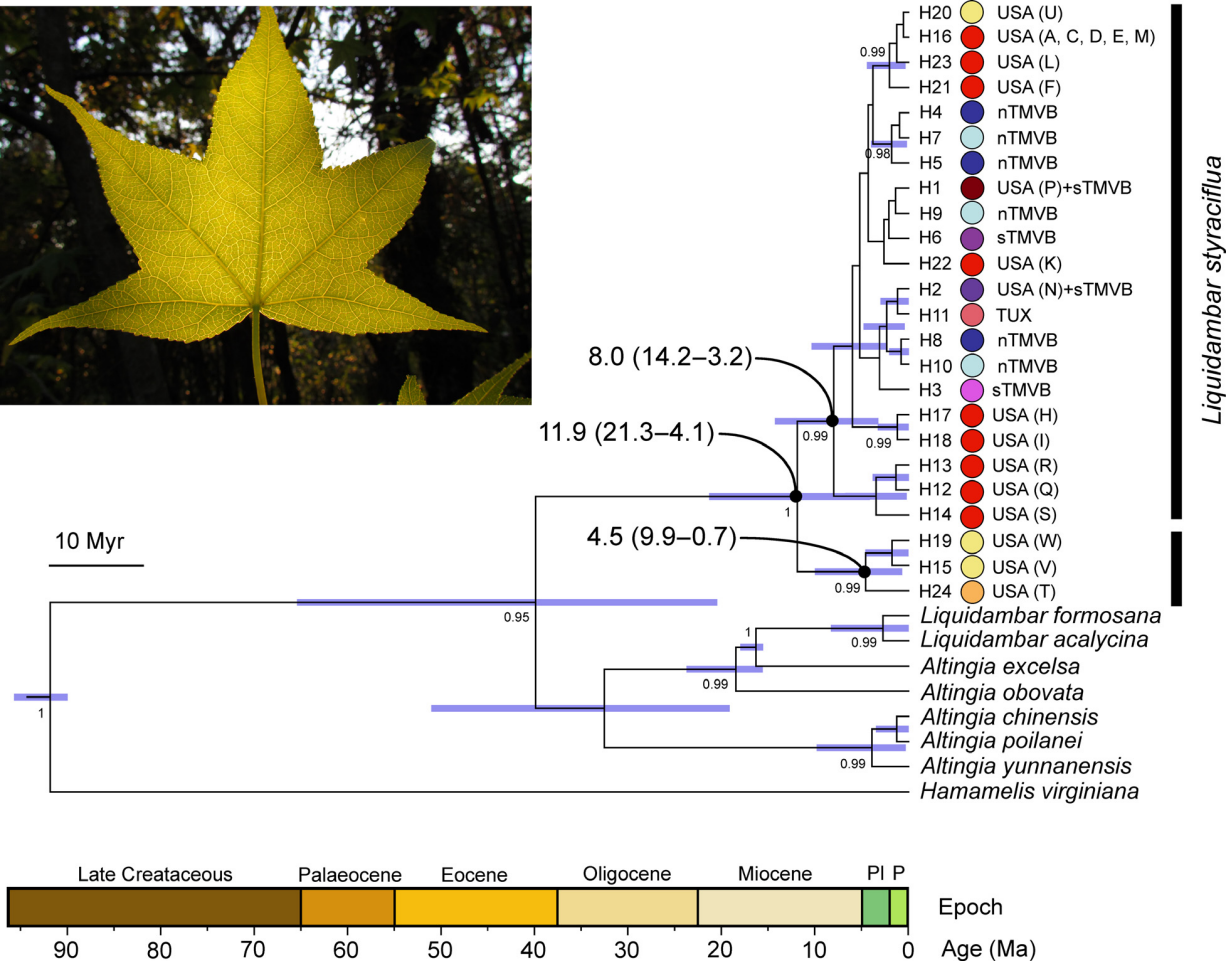
Chronogram of *Liquidambar styraciflua* haplotypes and other Altingiaceae based on the consensus tree from the Bayesian dating analysis using a coalescent model with constant size and the *psb*A*-trn*H/*psb*E-*pet*L combined dataset. For selected nodes, 95% Highest Posterior Density (HPD) intervals, indicated here by purple bars, and other inferred divergence estimates comparing different approaches are further summarized (in MYA) in the Results section. These nodes all have posterior probabilities of 0.95–1.0. The root of the tree was calibrated using *Microaltingia* Zhou, Crepet et Nixon (Zhou et al. 2001) from the Late Cretaceous of New Jersey. Ages in geological time are shown at the base of the tree. Color coding indicates major clusters of interest, and haplotype numbers are used in Fig. S1, which shows the statistical parsimony haplotype network and the geographic distribution of *Liquidambar styraciflua* cpDNA haplotypes in Mexico and the USA. Haplotype codes in parentheses (K–W) correspond to those in Morris et al. (2008).

The BEAST analysis suggests that the basal split between clade containing haplotypes H15, H19, and H24 (T–W in Morris et al. 2008) and other haplotypes of *L*. *styraciflua* occurred at ca. 11.9 MYA (95% HPD 21.3–4.2 MYA; Fig. 4). The age of the clade containing haplotypes Q–S (two individuals from Hackneyville, Alabama and one from Blaylock Mt., Arkansas) was 4.5 MYA (95% HPD, 9.9–0.7 MYA) and the US Gulf Coast clade (Morris et al. 2008) with the remaining haplotypes of *L. styraciflua* including those found in Mesoamerica was 8 MYA (95% HPD, 14.2–3.3 MYA).

### Genetic and phylogeographical structure

Differentiation among populations based on *psb*A*–trn*H/*psb*E–*pet*L variation (*G*_ST_ = 0.553, SE 0.0740) indicated that *L. styraciflua* is genetically highly subdivided, which is corroborated by the high fixation index (*F*_ST_) of 0.86. Genetic diversity (*h*_T_ = 0.959, SE 0.0112; *v*_T_ = 0.970, SE 0.0691) across all populations was higher than the average within-population (*h*_S_ = 0.428, SE 0.0688; *v*_S_ = 0.202, SE 0.0474), and, consequently, population differentiation was high across the entire range of the species in Mesoamerica. The level of *N*_ST_ (0.792, SE 0.0510) was significantly higher (*P *<* *0.05) than that for *G*_ST_, indicating phylogeographical structuring.

AMOVA results for data grouped as US and Mesoamerican populations revealed that 31.1% of the variation was explained by differences between groups, 8.8% by differences within populations, and 60.1% by differences between populations within groups (Table 1). When Mesoamerican populations were treated as a single group, 86.7% of the variation was explained by differences between populations. The AMOVA results revealed strong population structure with highest *F*_CT_ value obtained when samples were grouped by north and south of the TMVB and TUX region (*F*_CT_ = 0.4; Table 1). When sampling sites were grouped as separated by the TMVB (TUX included in the south of the TMVB group), a significant but smaller proportion of the variation was attributed to differences between groups (*F*_CT_ = 0.29; Table 1). SAMOVA results for the Mesoamerican locations revealed significant *F*_CT_ values for groups between *K* = 2 and *K* = 5, with the highest *F*_CT_ values for *K* = 3 (*F*_CT_ = 0.53; Table 1). The three groups identified correspond to populations 1–10 north of the TMVB, populations 12–13 in the TUX region, and populations south of the TMVB including population 11 in Veracruz (Chiconquiaco), 13–14 in Oaxaca (Comaltepec and Chayotepec), and 15–18 in Chiapas. When *K* = 4, *F*_CT_ is smaller than *K* = 3, and an additional increase in the number of *K* led to a dissolution of group structure.

**Table 1:** Results of AMOVA on *Liquidambar styraciflua* populations from USA (Morris et al. 2008) and Mesoamerica (a), and AMOVA and SAMOVA models on Mesoamerican populations with no groups defined a priori (b), by populations separated by the Trans-Mexican Volcanic Belt (c) and by genetic groups (populations separated by the Trans-Mexican Volcanic Belt and Los Tuxtlas) (d), and SAMOVA *K *= 3 groups (3).

	df	Sum of squares	Variance components	Percentage of variation	Fixation indices
(a) USA and Mesoamerica					
Among groups	1	77.977	0.5719	31.09	*F*_CT_ = 0.31***
Among populations within groups	44	286.419	1.1052	60.09	*F*_SC_ = 0.87***
Within populations	221	35.832	0.1621	8.82	*F*_ST_ = 0.91***
Total	226	400.228	1.8393		
(b) No groups defined					
Among populations	18	108.032	0.7053	86.77	
Within populations	141	15.166	0.1075	13.23	*F*_ST_ = 0.86***
Total	159	123.198	0.8128		
(c) Trans-Mexican Volcanic Belt					
Among groups	1	28.034	0.2826	29.90	*F*_CT_ = 0.29*
Among populations within groups	17	79.998	0.5549	58.72	*F*_SC_ = 0.83***
Within populations	141	15.166	0.1075	11.38	*F*_ST_ = 0.88***
Total	159	123.198	0.9452		
(d) Genetic groups					
Among groups	2	44.925	0.3963	40.95	*F*_CT_ = 0.40**
Among populations within groups	16	66.106	0.4640	47.94	*F*_SC_ = 0.81***
Within populations	141	15.166	0.1075	11.11	*F*_ST_ = 0.88***
Total	159	123.198	0.9680		
(e) SAMOVA *K* = 3					
Among groups	2	114.642	1.1307	53.03	*F*_CT_ = 0.53***
Among populations within groups	16	86.685	0.6078	28.51	*F*_SC_ = 0.60***
Within populations	141	55.517	0.3937	18.46	*F*_ST_ = 0.81***
Total	159	256.844	2.1323		

* ^*^P <* *0.01

** *P *<* *0.001

*** *P *<* *0.0001.

### Historical demography

Patterns of demographic history inferred for Mesoamerican populations of *L. styraciflua* are somewhat ambiguous. Small, mostly negative Fu's *Fs* and Tajima's *D* values indicate that TUX or groups separated by the TMVB have not undergone expansion (Table S2). In the mismatch distribution, sudden demographic expansion (SSD and Hri values) was not rejected for regional groups (Table S2). The BSPs of *Ne* through time were flat across time, with no significant increase in population size in the groups north and south of the TMVB postdating the LGM (Fig. S2).

IMa results are summarized in Table 2 and Figs S3–S4. Results are reported as highest point estimates and 95% highest probability density (HPD). Based on the mutation rates of 1.0 × 10^−9^, 1.59 × 10^−9^, and 3.0 × 10^−9^ s/s/y, the ancestral population size (*N*_A_) is estimated to be higher than population sizes of descendant populations for both USA/Mesoamerican and north/south of TMVB splits (Table 2 and Figs S3–S4). We obtained single narrow peaks for the posterior probability distributions of all the parameters, but the tail of the posterior distribution of the effective population size of the ancestral population with descendant populations north and south of the TMVB did not reach zero over a large range of priors as the other parameters (Fig. S4). Divergence between the US and Mesoamerican populations occurred at 8.4 MYA based on the low mutation rate and 2.8 MYA based on the high mutation rate, and 4.3 MYA and 1.4 Ma between populations located north and south of the TMVB for low and high mutation rates, respectively. Based on the intermediate mutation rate (1.59 × 10^−9^ s/s/y), the divergence between the US and Mesoamerican populations occurred at 5.3 MYA, and 2.7 Ma between populations located north and south of the TMVB. When testing for migration following the split between US versus Mesoamerican populations, extremely low-effective migration rates (close to zero) from USA to Mesoamerica are indicated with higher migration in the reverse direction. Effective migration following the split between populations north and south of the TMVB was close to zero in both directions, suggesting that the unsorted tree we observed is the result of incomplete lineage sorting and incomplete coalescence and not due to significant levels of gene flow across the TMVB (Table 2).

**Table 2:** Results of isolation-with-migration model (IMa) for the splits USA versus Mesoamerica and north versus south of the TMVB based on chloroplast sequences. Model parameters indicate estimates without use of molecular rate of evolution for six parameters (IMa output values). Demographic rates represent parameters scaled to rate of molecular evolution (1.0 × 10^−9^ s/s/y, 3.0 × 10^−9^ s/s/y, 0.3 × 10^−9^ s/s/y); *q* parameters in thousands of effective population size (*N*_e_), *m* in genes per generation of effective migration rate (*N*m), *t* parameter in millions of years.

Model parameter estimates	*q*_1_	*q*_2_	*q*_A_	*m*_1_	*m*_2_	*t*
USA vs. Mesoamerica	6.919	18.661	198.785	0.134	0.715	11.899
HPD 95%	3.083–12.489	9.788–31.87	7.973–398.082	0.003–0.435	0.211–1.535	2.712–24.087
North vs. south of the TMVB	6.333	3.826	3527.922	0.116	0.172	6.057
HPD 95%	2.397–12.668	0.940–9.261	311.180–9585.365	0.002–0.501	0.003–0.726	1.070–17.770

Demographic parameter estimates	Mutation rate	Generation time	*N*_1_	*N*_2_	*N*_A_	Nm_1_	Nm_2_	*t*
USA vs. Mesoamerica	1.0 × 10^−9^	34	71,806	193,666	2,063,026	0.4635	6.6713	8,397,318
HPD 95%			31,994–129,612	101,580–330,752	82,744–4,131,366	0.0046–2.7163	1.0326–24.4602	1,913,902–16,998,588
		124	19,688	53,102	565,668			
			8,772–35,538	27,852–90,690	22,688–1,132,794			
	1.59 × 10^−9^	34	45,163	121,808	1,297,552			5,281,402
			20,124–81,520	63,890–208,028	52,043–2,598,446			1,203,728–10,691,078
		124	12,383	33,398	355,773			
			5,517–22,352	17,517–57,039	14,269–712,463			
	3.0 × 10^−9^	34	23,934	64,544	687,674			2,799,106
			10,664–43,204	33,860–110,250	27,580–1,377,122			637,967–5,666,196
		124	6,562	17,700	188,556			
			2,924–11,846	9,284–30,230	7,562–377,598			
North vs. south of the TMVB	1.0 × 10^−9^	34	65,724	39,706	36,613,412	0.3701	0.3298	4,269,583
HPD 95%			24,876–131,470	9,754–96,118	3,229,482–99,478,652	0.0029–3.1765	0.0016–3.3643	755,116–12,540,578
		124	18,020	10,886	10,039,160			
			6,820–36,048	2,664–26,352	885,502–27,276,404			
	1.59 × 10^−9^	34	41,338	24,972	23,028,211			2,688,415
			15,646–82689	6,140–60,450	2,031,201–62,567,656			474,922–7,887,261
		124	11,334	6,847	6,314,067			
			4,290–22,672	1,682–16,574	556,931–17,155,322			
	3.0 × 10^−9^	34	21,908	13,234	12,204,470			1,423,124
			8,292–43,822	3,250–32,036	1,076,494–33,159,550			251,705–4,180,192
		124	6,006	3,628	2,146,386			
			2,272–12,016	890–8,784	295,166–9,092,134			

### Hypothesis testing of Pleistocene refugia

We calculated *s *=* *212 (Slatkin and Maddison 1989) for our Bayesian genealogy. Simulations at the highest *N*e value (1 × 10^6^) for the time as the most recent glacial advance (T4: 120,000 years BP) rejected the two-refugia hypothesis but not the fragmentation hypothesis using a generation time of 34 years (Table S3). For other *N*e values, both the two-refugia and fragmentation hypotheses were rejected at any divergence time (T1–T3). This pattern upheld when considering a generation time of 124 years except that both hypotheses were not rejected at low divergence time values (T3 and T4; Table S3). Thus, our coalescent simulations support the null hypothesis of the current disjunct distribution being the result of fragmentation of a single widespread ancestral lineage.

### Ecological niche modeling

**Figure 5:**
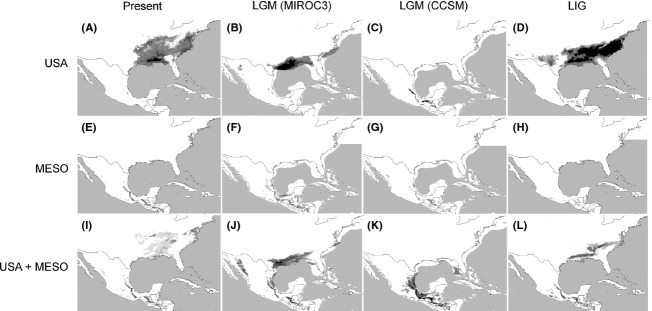
Results from the MAXENT analyses showing species distribution models for *Liquidambar styraciflua* for USA distribution (records in Mexico excluded; A–D), Mesoamerican distribution (records in USA excluded; E–H), and full distribution in USA and Mesoamerica (I–L) at present, Last Glacial Maximum (LGM, MIROC), Last Glacial Maximum (LGM, CCSM), and Last Interglacial (LIG). Darker shading indicates the highest predicted probability of occurrence. There is clear evidence that USA and Mesoamerican populations and populations within Mesoamerica are not connected during glacial cycles.

The current distribution predicted by MAXENT (Fig. 5) closely matched the known range of *L. styraciflua*, and the models performed well (all AUC values >0.948). The ENM for the current climate variables using both the US and Mesoamerican records predicted well the Mesoamerican distribution and under predicted some known localities in the USA (Fig. 5). When our models were projected onto past climatic layers, we obtained two different scenarios. For the climate layers based on MIROC, we predicted a large southern area of suitable habitat across the southeastern USA and a second area in Mexico with a large geographic disjunction at the Isthmus of Tehuantepec. There was some over prediction in the Sierra Madre Occidental when using US and Mesoamerican records (Fig. 5). Predictions based on CCSM suggest that suitable habitat for *L. styraciflua* in the USA was almost absent, with the potential suitable habitat of US populations restricted to the Florida Peninsula and the distribution of habitat suitability of Mesoamerican populations connected and expanded to lower coastal areas along the Gulf of Mexico. Lastly, models projected onto LIG climatic layers (120–140K) revealed a similar fragmented scenario to the current ENM predicted, with a reduction in suitable habitat in eastern Mexico when using the Mesoamerican dataset. As predicted by the palaeodistributional pattern, both the US and Mesoamerican populations persisted disjunct in their present ranges through the later Pleistocene rather than a pattern of expansion to the south.

The PCA of environmental data indicated three niche axes that together explained 95.2% of the variation in *L. styraciflua* (US and Mesoamerican records). The first niche axis (62.1% of variation) was associated with temperature. The second niche axis (27.7%) was associated with annual precipitation and precipitation of coldest quarter, whereas the third axis (5.4%) was associated with coldest months. Tests of niche divergence and conservatism on these three niche axes showed evidence for niche conservatism on niche axis 1 and 2 (89.8%; Table 3). In contrast, evidence of niche divergence is shown for the Mesoamerican records (north and south of the TMVB). The first niche axis (55.2%) was associated with precipitation variables, whereas niche axes 2 (22.2%), 3 (12.3%), and 4 (5.4%) were associated with temperature. Tests of niche divergence and conservatism of these four axes showed evidence of niche divergence on axes 1 and 3 (67.5%; Table 3).

**Table 3:** Loadings of the environmental variables for each PC axis and tests of niche divergence and conservatism. Observed differences in climatic niche of *Liquidambar* lineages on each PC compared with the middle 95th percentile of a null distribution of the differences between their environmental backgrounds. (a) USA vs. Mesoamerica) and (b) nTMVB vs. sTMVB.

	PC1	PC2	PC3	PC4
(a) USA vs. Mesoamerica				
BIO6: min temperature of coldest month	0.3749	−0.0424	0.4438	–
BIO7: temperature annual range	−0.3753	0.0597	−0.2720	–
BIO11: mean temperature of coldest quarter	0.3746	−0.0567	0.4234	–
BIO12: annual precipitation	0.2507	0.4573	−0.0350	–
BIO13: precipitation of wettest month	0.3456	0.2657	−0.1738	–
BIO14: precipitation of driest month	−0.2924	0.3942	0.1307	–
BIO16: precipitation of wettest quarter	0.3406	0.2820	−0.1564	–
BIO17: precipitation of driest quarter	−0.2927	0.3993	0.1548	–
BIO18: precipitation of warmest quarter	0.2390	0.3640	−0.4492	–
BIO19: precipitation of coldest quarter	−0.2275	0.4300	0.5012	–
Percent variance explained	62.12	27.67	5.45	–
Observed difference	4.3787*	0.0880*	0.0828*	–
Null distribution	4.7672–4.7692	0.6518–0.6537	0.04608–0.04686	–
(b) nTMVB vs. sTMVB				
BIO6: min temperature of coldest month	0.1077	0.6253	0.2015	0.1418
BIO7: temperature annual range	−0.0965	−0.4158	0.5409	0.5188
BIO11: mean temperature of coldest quarter	0.0760	0.6018	0.3234	0.2078
BIO12: annual precipitation	0.4148	0.0056	0.0555	−0.2517
BIO13: precipitation of wettest month	0.3674	−0.1445	0.2864	−0.3433
BIO14: precipitation of driest month	0.3768	−0.1209	−0.2465	0.3762
BIO16: precipitation of wettest quarter	0.3807	−0.0864	0.2551	−0.3914
BIO17: precipitation of driest quarter	0.3841	−0.0765	−0.2722	0.3503
BIO18: precipitation of warmest quarter	0.3296	−0.1073	0.3512	0.2229
BIO19: precipitation of coldest quarter	0.3510	0.1162	−0.3923	0.1388
Percent variance explained	55.26	22.22	12.29	5.36
Observed difference	0.4049*	2.0390*	0.7765*	0.2985*
Null distribution	0.3499–0.3597	2.2718–2.2749	0.5267–0.5307	0.3841–0.3862

nTMVB, north of the Trans-Mexican Volcanic Belt, sTMVB, south of the Trans-Mexican Volcanic Belt.

Bold values indicate niche conservatism.

* Significance level, *P *<* *0.05.

## Discussion

### Phylogeographic structure in Mesoamerica

Morris et al. (2008) sampled *L. styraciflua* from many localities in the USA, with limited sampling in Mesoamerica. We integrated ecological niche modeling, historical demography, gene flow estimates, coalescent tests, and coalescent-based estimation of intraspecific divergence times to address the same phylogeographical system, sampling more localities and individuals within Mesoamerica, while attempting to reconstruct the dynamic biogeographical history for this cloud forest tree species. Our results showed that the Mesoamerican sweetgum is composed of three haplogroups formed by private haplotypes. Most of the haplotypes that formed the first group (H1–H11) were only found in the cloud forests of the Sierra Madre Oriental north of the TMVB, haplotypes H15–H17 formed the second group of private haplotypes corresponding to the TUX region, and the remaining private haplotypes (except H20) clustered in a third group south of the TMVB. This pattern of the haplotype network was also supported by the AMOVA and SAMOVA analyses. However, patterns of haplotype sharing between the groups separated by the TMVB suggest historical gene flow, as haplotype H20, which is located in populations north of the TMVB, is descendant of haplotype H19, from south of the TMVB. Nonetheless, gene flow close to zero among populations on both directions of the TMVB was confirmed by the IMa results, supporting the idea that the TMVB is a porous barrier. Lastly, the private haplotypes retrieved in the populations of the TUX region are indicative of allopatric fragmentation and constitute genetically unique populations. Conservation of the TUX region is particularly important due to the restricted distribution of the cloud forest, and because of the accelerated deforestation rates in the TUX region that threaten the endemic genetic diversity of these and possibly other codistributed taxa (Ornelas et al. 2013).

### The American sweetgum in Mesoamerica

Using an integrative analytical approach, we were able to identify evolutionary divergences between disjunctions across the range of *L. styraciflua* and to determine whether or not its current distribution is associated with pre-Quaternary climatic events. Phylogeographical patterns, asymmetrical gene flow patterns, and coalescent-based divergence time inference of *L. styraciflua* support a Miocene arrival of the temperate element into Mexico (Braun 1950), a scenario consistent with fossil data (Graham 1999), and the presence of *Liquidambar* in the microfossil flora of the Paraje Solo formation in southeastern Veracruz (Graham 1993). The observed palaeodistribution pattern of our ecological niche modeling supports this scenario in which both the US and Mesoamerican populations persisted disjunct in their present ranges through the later Pleistocene rather than a pattern of expansion to the south during glacial cycles. The disjunct distribution might have resulted from vicariance or long-distance dispersal, or from a combination of both. In the present study, cpDNA sequence data support long-distance dispersal because Mesoamerican lineages were nested within those from the USA (not-reciprocally monophyletic; see also Wang et al. 2013 and Zhao et al. 2013). The statistical parsimony network of US and Mesoamerican samples (Fig. S1), and the phylogenetic results of MrBAYES and BEAST were not fully resolved into two distinct groups, indicating the clade from the highlands of the Cumberland Plateau and the Southern Appalachians as monophyletic (see also Morris et al. 2008), while paraphyly was observed in the group along the US Gulf Coast and Mesoamerica. However, the high levels of genetic diversity (*h*_T_ = 0.959, *v*_T_ = 0.970) within a presumably recently colonized area (Mesoamerica) are not expected under the long-distance dispersal model. Considering the high haplotype and genetic diversity observed in Mesoamerican populations, a vicariance scenario should be the most likely explanation and nonreciprocal monophyly of the US and Mesoamerican populations likely the result of incomplete lineage sorting due to insufficient time. Nonetheless, our coalescent simulations support the null hypothesis of the current disjunct distribution being the result of fragmentation of a single widespread ancestral lineage since the most recent glacial advance. The coalescent simulations used in this study provide a means of inferring population history even in the face of incomplete lineage sorting by assessing the stochasticity inherent in the coalescent process (Edwards and Beerli 2000; Spellman and Klicka 2006). However, the results of our simulations must be taken with caution because rely upon population parameters inferred from single locus data.

The US and Mesoamerican genetic break of *L. styraciflua* is hypothesized to result from climatic warming and drying during the late Miocene to early Pleistocene (Graham 1999). The divergence times estimated in IMa (and BEAST) is corroborated with the palynological data which indicated that the Tertiary floras in southern Mexico contained *Liquidambar* pollen in the early-to-middle Miocene (Chávez 1993; Graham 1993, 1999; Martínez-Hernández and Ramírez-Arriaga 1999), and dates for the same split between northeastern Texas and northeastern Mexico (southern Tamaulipas) populations of *Smilax hispida* and *S. jalapensis* (Zhao et al. 2013) and *Quercus virginiana* and *Q. oleoides* (Cavender-Bares et al. 2011). These results are in contrast to those previously found in *L. styraciflua* (Morris et al. 2008) and *Hamamelis* (*H. virginiana* and *H. mexicana*; Xie et al. 2010), in which the Mexican species/populations were found to have a Pleistocene origin from eastern North America. Furthermore, our palaeodistribution modeling does not support disjunct Pleistocene refugia in *L. styraciflua*, suggesting that, while populations may have retreated into coastal refugia, the disjunction was probably not driven by expansion/contraction during glacial/interglacial cycles but earlier patterns of climate change. The pattern of haplotype variation for the combined US and Mesoamerican samples (Fig. S1) suggests a Mesoamerican colonization hypothesis for *L. styraciflua* in the USA, although phylogenetic relationships that would clarify this remain unresolved (see also Morris et al. 2008). The most widespread haplotype (H1; Fig. S1) found south of the TMVB and extending to the USA is the more central and potentially the most ancestral haplotype in the network. Most of the external haplotypes are only found in specific regions (USA, nTMVB, sTMVB, TUX) and are therefore likely to be more recent. The Mesoamerican colonization hypothesis is also consistent with the lower (close-to-zero) migration rates of US populations southwards than of Mesoamerican populations northwards (Table 2; Figs 4), and by the results of the tests of niche conservatism that suggest that the Mesoamerican colonization with greater gene flow northwards was facilitated by niche similarity (Table 3).

### The Trans-Mexican Volcanic Belt

The Trans-Mexican Volcanic Belt (TMVB) is a volcanic chain of mountains that virtually cuts Mexico into northern and southern halves, and its geological development had a profound impact as a biogeographical barrier between Nearctic and Neotropical biotas (Halffter 1987; Marshall and Liebherr 2000; Mulcahy et al. 2006; Morrone 2010; Bryson et al. 2011). Recent phylogeographical studies have shown that the uplift of the TMVB is correlated with the diversification of bunchgrass lizards (*Sceloporus scalaris*; Bryson et al. 2012), salamanders (*Pseudoeurycea leprosa*; Parra-Olea et al. 2012), and the shrub *Nolina parviflora* (Ruiz-Sanchez and Specht 2013) indicating that habitat diversity within the TMVB may drive speciation in these lineages. Neogene vicariance appears to be the primary driver of diversification, and the divergences were temporally and geographically congruent with distinct periods of uplift across the TMVB (Gómez-Tuena et al. 2007; reviewed in Bryson et al. 2011). However, to date, no phylogeographical study of plant species has shown that the TMVB has been a geographic barrier to gene flow between northern and southern populations. Sosa et al. (2009) analyzed the phylogeographic structure of the xerophytic perennial herb *Hunnemannia fumariifolia* (Papaveraceae) in eastern Mexico, and the authors found genetic differentiation between populations south of the TMVB (Tehuacán-Cuicatlán Valley) from those north of the volcanic belt (Sierra Madre Oriental and the Chihuahuan Desert), and the ENM reconstructions agreed with a scenario of expansion south to north of the TMVB during the LGM (Ruiz-Sanchez et al. 2012).

Our phylogeographic analyses highlight the importance of the TMVB in driving isolation and genetic divergence between *L. styraciflua* populations distributed north and south of the volcanic belt. Substitution rates of 1.0 × 10^−9^, 1.59 × 10^−9^, and 3.0 × 10^−9^ s/s/y indicated that divergence of populations separated by the TMVB occurred during the Pliocene to mid-Pleistocene (4.27, 2.68 and 1.42 MYA, respectively). The most likely explanation for this disjunction is one of the most recent episodes of the geologic evolution of the TMVB, the formation of the Pliocene to Quaternary volcanic arc that occurred at ca. 3.6 MYA, and subsequent climatic changes in the region (Gómez-Tuena et al. 2007). The TMVB is a region of extreme elevational changes within relatively short distances, with a corresponding diversity of habitats (Parra-Olea et al. 2012). Consequently, following geographic isolation by the TMVB, the populations of *L. styraciflua* separated by the volcanic belt would have been exposed and eventually adapted to their differing environmental conditions. Populations of the Mesoamerican sweetgum separated by the TMVB are distributed in unique environmental space, implying that the differing environmental conditions of on either side of the TMVB would have reduced gene flow, as shown by IMa results, that would have reinforced the divergence of the two cpDNA haplogroups following initial spatial separation (see also Liu et al. 2013). This scenario is supported by tests of niche divergence and conservatism that compared the amount of climatic divergence to the null expectation of background climatic divergence and showed evidence for niche divergence between sweetgum records north and south of the TMVB on two axes of environmental space related to precipitation (PC1). These findings support the hypothesis that climatic niche dissimilarity between sweetgum populations separated by the TMVB seems to have reduced gene flow. Our analyses of *L. styraciflua*, combining a phylogeographic and species distribution modeling approach, suggest that the observed patterns of genetic variation and divergence in Mesoamerica are best explained by a combination of isolation imposed by the TMVB and reinforcement by subsequent differential climatic conditions. Although the latter may be true for species that disperse poorly or that are reluctant for physiological reasons to cross regions of less-hospitable habitat, niche divergence for other species with poor seed dispersal may equate to enhanced opportunities for isolation and reduced gene flow, thereby increasing the likelihood of speciation.

## Data Accessibility

DNA sequences: GenBank accession nos KF009884–KF009912, KF009913–KF009941. IMa input files deposited in the Dryad Repository: http://dx.doi:10.5061/dryad.t36v1. Voucher specimens (see Table S1) are archived at the Herbarium of the Instituto de Ecología, AC (XAL).

## Supporting Information

Data S1 Sequencing protocols, Bayesian inference protocols, and outgroup justification.

**Figure S1.** Geographic distribution and statistical parsimony haplotype network of *Liquidambar styraciflua* cpDNA haplotypes including our samples from Mexico and those in the USA from Morris et al. (2008). **Figure S2.** Bayesian skyline semilog plots showing medians for the historical demographic trends of *Liquidambar styraciflua* on the north (top) and the south (bottom) side of the Trans-Mexican Volcanic Belt. **Figure S3.** Posterior probability distributions of parameters calculated in IMa of Mesoamerican and US populations. **Figure S4.** Posterior probability distributions of parameters calculated in IMa of Mesoamerican populations separated by the Trans-Mexican Volcanic Belt.

**Table S1.** Geographic location and population code of the 19 *Liquidambar styraciflua* populations used in the study. **Table S2.** Summary statistics of demographic analysis of *Liquidambar styraciflua* grouping samples by (a) populations and (b) groups separated by Trans-Mexican Volcanic Belt to infer demographic range expansion. **Table S3.** Summary of coalescent analyses of various models of population structure of *Liquidambar styracifua*.
