# Supplementary material for: Phylogeography of Liquidambar styraciflua (Altingiaceae) in Mesoamerica: survivors of a Neogene widespread temperate forest (or cloud forest) in North America?
Source: Ecol Evol. 2014 Jan 10;4(4):311–28. doi: 10.1002/ece3.938 (PMC3936380; doi:10.1002/ece3.938)
Supplement: Data S1 — Sequencing protocols, Bayesian inference protocols, and outgroup justification. [file ece30004-0311-sd1.doc]

**Supporting Information**

Phylogeography of *Liquidambar styraciflua* (Altingiaceae) in Mesoamerica:

survivors of a Neogene widespread temperate forest (or cloud forest) in North America?

Eduardo Ruiz-Sanchez and Juan Francisco Ornelas

*Ecology and Evolution*

**Data S1.** Sequencing protocols, Bayesian inference protocols and outgroup justification.

**Materials and Methods**

**Sequencing protocols**

Total genomic DNA from silica-dried material was extracted using a modified 2 × cethyl trimethyl ammonium bromide (CTAB) protocol (Doyle and Doyle 1987). The chloroplast *psb*A*–trn*H plastid region was amplified using the primers in Sang et al. (1997) and Tate and Simpson (2003). The chloroplast *psb*E–*pet*L plastid region was amplified using the primers in Morris et al. (2008). PCR reactions (15 µL total volume) contained 3µL of 5× buffer, 1.5μL of 25 mM MgCl_2_, 1.2µL of 8 mM of dNTPs mix, 0.6–1µL of 10 µM of each primer, 0.12µL of 5U/µL of Taq polymerase (Promega, Madison, WI, USA), 0.2–0.5 of BSA, and 1.5–3µL of DNA. PCR reactions were performed in a 2720 thermal cycler (Applied Biosystems, Carlsbad, CA, USA) with the following temperature profile: initial denaturing step at 80°C for 5 min, 35 cycles of 94°C for 30 s, 50°C for 30 s, and 75°C for 1 min followed, and a final extension of 72°C for 10 min. PCR products were purified with the QIAquick kit (Qiagen, Valencia, CA, USA) and sequenced in both directions to check the validity of sequence data using the BigDye Terminator Cycle Sequencing kit (Applied Biosystems, Ann Arbor, MI, USA). Sequences were read in a 310 automated DNA sequencer (Applied Biosystems) at the Instituto de Ecología, AC sequencing facility, or at htSEQ high Troughtput Sequencing, Washington, USA or at Macrogen, Seoul, South Korea.

**Relationships among haplotypes**

For the Bayesian Inference (BI) analysis we allowed four incrementally heated Markov chains to proceed for 10 million generations, sampling every 1000 generations. Bayesian posterior probability values were calculated from the sampled trees remaining after 10% of burn-in (Huelsenbeck and Ronquist 2001) samples were discarded to only include trees after stationarity was reached. A 50% majority-rule consensus tree was made, showing nodes with a posterior probability (PP) of 50% or more. We considered those nodes with ≥ 95% Bayesian posterior probabilities as strongly supported (Huelsenbeck and Ronquist 2001). We rooted characters using the outgroup method on the concatenated data set. While *L. styraciflua* and *L. orientalis* may be close relatives (Ickert-Bond and Wen 2006; Ozdilek et al. 2012), relationships among these *Liquidambar* species remain uncertain (Morris et al*.* 2008). Thus, *psb*A*–trn*H/*psb*E–*pet*L sequences of *L. orientalis* (EU595855–EU595864) of a specimen from the University of Washington Arboretum (voucher WA 1386-56) were not included because the sample may not be true *L. orientalis* (Ozdilek et al. 2012). We obtained GenBank cpDNA sequences of *Hamamelis virginiana* (EU595863–EU595872), *Altingia chinensis* (EU595856–EU595865), *A. obovata* (EU595862–EU595871), *A. poilanei* (EU595858–EU595867), *A. yunnanensis* (EU595857–EU595866), *Liquidambar formosana* (EU595861–EU595870), *L. acalycina* (EU595860–EU595869), and *L. styraciflua* of southeastern USA populations from Morris et al. (2008) to root our *L. styraciflua* sequences.

**References**

Doyle, J. J., and J. L. Doyle. 1987. A rapid DNA isolation procedure from small quantities of fresh leaf tissue. Phytochem. Bull. 19:11–15.

Huelsenbeck, J. P., and F. Ronquist. 2001. MRBAYES: Bayesian inference of phylogeny. Bioinformatics 17:754–755.

Ickert-Bond, S. M., and J. Wen. 2006. Phylogeny and biogeography of Altingiaceae: evidence from combined analysis of five non-coding chloroplast regions. Mol. Phylogenet. Evol. 39:512–528.

Morris, A. B., S. M. Ickert-Bond, B. Brunson, D. E. Soltis, and P. S. Soltis. 2008. Phylogeographical structure and temporal complexity in American sweetgum (*Liquidambar styraciflua*; Altingiaceae). Mol. Ecol. 17:3889–3900.

Ozdilek, A., B. Cengel, G. Kandemir, Y. Tayanc, E. Velioglu, and Z. Kaya. 2012. Molecular phylogeny of relict-endemic *Liquidambar orientalis* Mill based on sequence diversity of the chloroplast-encoded *mat*K gene. Pl. Syst. Evol. 298:337–349.

Sang, T., D. J. Crawford, and T. F. Stuessy. 1997. Chloroplast DNA phylogeny, reticulate evolution, and biogeography of *Paeonia* (Paeoniaceae). Am. J. Bot. 84:11201136.

Tate, J. A., and B. B. Simpson. 2003. Paraphyly of *Tarasa* (Malvaceae) and diverse origins of the polyploidy species. Syst. Bot. 28:723–737.
