# Supplementary material for: Phylogeography of Liquidambar styraciflua (Altingiaceae) in Mesoamerica: survivors of a Neogene widespread temperate forest (or cloud forest) in North America?
Source: Ecol Evol. 2014 Jan 10;4(4):311–28. doi: 10.1002/ece3.938 (PMC3936380; doi:10.1002/ece3.938)
Supplement: Supplementary file 2 — Figure S1. Geographic distribution and statistical parsimony haplotype network of Liquidambar styraciflua cpDNA haplotypes including our samples from Mexico and those in the USA from Morris et al. (2008). Figure S2. Bayesian skyline semilog plots showing medians for the historical demographic trends of Liquidambar styraciflua on the north (top) and the south (bottom) side of the Trans-Mexican Volcanic Belt. Figure S3. Posterior probability distributions of parameters calculated in IMa of Mesoamerican and US populations. Figure S4. Posterior probability distributions of parameters calculated in IMa of Mesoamerican populations separated by the Trans-Mexican Volcanic Belt. [file ece30004-0311-sd2.doc]

**Supporting Information**

Phylogeography of *Liquidambar styraciflua* (Altingiaceae) in Mesoamerica:

survivors of a Neogene widespread temperate forest (or cloud forest) in North America?

Eduardo Ruiz-Sanchez and Juan Francisco Ornelas

*Ecology and Evolution*

**Figure S1.** Geographical distribution and statistical parsimony haplotype network of *Liquidambar styraciflua* cpDNA haplotypes including our samples from Mexico and those in the USA from Morris et al. (2008). Pie charts represent the haplotypes found in each sampling locality. The size of sections of the pie charts is proportional to the number of individuals with that haplotype, and small filled circles are non-sampled haplotypes. The numbers in the haplotypes indicate the number of individuals that share that haplotype.

**Figure S2.** Bayesian skyline semilog plots showing medians for the historical demographic trends of *Liquidambar styraciflua* on the north (top) and the south (bottom) side of the Trans-Mexican Volcanic Belt. Along the y-axis the population size is estimated in units of *N*_e_μ (*N*_e_: effective population size, l: mutation rate per haplotype per generation). The x-axis shows the calendar time converted into thousands of years ago using the mutation rates of 1.0 × 10^–9^ (left) and 3.0 × 10^–9^ (right) substitutions/site/year. Solid lines represent median estimates and shaded areas represent 95% confidence intervals.

**Figure S3.** Posterior probability distributions of parameters calculated in IMa. (a) *q*_A_, (b) *q*_N_, (c) *q*_S_ are the ancestral, Mesoamerican (M) and US (U) population sizes, respectively; (d) *m*_S–N_, (e) *m*_N–S_ are the migration rates south-to-north (U-M) and north-to-south (M-U), respectively; (f) *t* is the time since divergence. IMa runs yielded marginal distributions of *t* with a single, sharp peak of high probability followed by a long tail of considerably lower probability that never dropped to zero. This result can be viewed as minimum (most recent) estimates of divergence time, with some probability of later divergence, as the parameter estimate is non-zero for later (earlier) divergence times.

**Figure S4.** Posterior probability distributions of parameters calculated in IMa. (a) *q*_A_, (b) *q*_N_, (c) *q*_S_ are the ancestral, northern and southern population sizes, respectively; (d) *m*_S–N_, (e) *m*_N–S_ are the migration rates south-to-north and north-to-south over the Trans-Mexican Volcanic Belt, respectively; (f) *t* is the time since divergence. IMa runs yielded marginal distributions of *q*_A_ with a single, sharp peak of high probability followed by a long tail of considerably lower probability that never dropped to zero. This result can be viewed as minimum estimates of ancestral population size, with some probability of larger size, as the parameter estimate is non-zero for larger population sizes.
