# Supplementary material for: Phylogeography of Liquidambar styraciflua (Altingiaceae) in Mesoamerica: survivors of a Neogene widespread temperate forest (or cloud forest) in North America?
Source: Ecol Evol. 2014 Jan 10;4(4):311–28. doi: 10.1002/ece3.938 (PMC3936380; doi:10.1002/ece3.938)
Supplement: Supplementary file 3 — Table S1. Geographic location and population code of the 19 Liquidambar styraciflua populations used in the study. Table S2. Summary statistics of demographic analysis of Liquidambar styraciflua grouping samples by (a) populations and (b) groups separated by Trans-Mexican Volcanic Belt to infer demographic range expansion. Table S3. Summary of coalescent analyses of various models of population structure of Liquidambar styracifua. [file ece30004-0311-sd3.doc]

**Supporting Information**

Phylogeography of *Liquidambar styraciflua* (Altingiaceae) in Mesoamerica:

survivors of a Neogene widespread temperate forest (or cloud forest) in North America?

Eduardo Ruiz-Sanchez and Juan Francisco Ornelas

*Ecology and Evolution*

**Table S1.** Geographic location and population code of the 19 *Liquidambar styraciflua* populations used in the study.

| Location Code | Location | Region | *n* | Altitude  (m asl) | Latitude  (N) | Longitude (W) | Haplotype |
| --- | --- | --- | --- | --- | --- | --- | --- |
|  |  |  |  |  |  |  |  |
| 1 | San Luis Potosí, Aquismón | nTMVB | 2 | 378 | 21° 37´ 55´´ | 99° 03´ 34´´ | H20(2) |
| 2 | San Luis Potosí, Xilitla | nTMVB | 9 | 637 | 21° 22´ 39´´ | 98° 59´ 35´´ | H2(3), H5(6) |
| 3 | Hidalgo, San Bartolo Tutotepec | nTMVB | 14 | 1155 | 20° 21´ 11´´ | 98° 13´ 10´´ | H2(9), H3(1), H5(4) |
| 4 | Hidalgo, Tlanchinol | nTMVB | 11 | 1476 | 20° 56´ 55´´ | 98° 04´ 12´´ | H6(10), H7(1) |
| 5 | Querétaro, Sierra Gorda, Neblinas | nTMVB | 4 | 1130 | 21° 15´ 10´´ | 99° 04´ 31´´ | H5(2), H8(2) |
| 6 | Veracruz, Huayacocotla, Helechales | nTMVB | 9 | 1950 | 20° 37´ 17´´ | 98° 27´ 49´´ | H2(1), H5(7), H10(1) |
| 7 | Puebla, Huauchinango, Lagunillas | nTMVB | 6 | 1500 | 18° 12´ 49´´ | 98° 02´ 19´´ | H5(2), H8(1), H9(1), H12(2) |
| 8 | Puebla, Huitzilan | nTMVB | 13 | 975 | 19° 57´ 29´´ | 97° 41´ 17´´ | H1(7), H2(2), H4(1) |
| 9 | Veracruz, Coatepec, La Cortadura | nTMVB | 10 | 2100 | 19° 29´ 05´´ | 97° 02´ 20´´ | H11(1), H12(6), H13(2), H14(1) |
| 10 | Veracruz, Huatusco | nTMVB | 10 | 1215 | 19° 11´ 07´´ | 96° 57´ 32´´ | H12(10) |
| 11 | Veracruz, Chiconquiaco | nTMVB | 5 | 2005 | 19° 44´ 58´´ | 96° 48´ 54´´ | H18(5) |
| 12 | Veracruz, San Andrés Tuxtla | TUX | 10 | 648 | 18° 28´ 46´´ | 95° 10´ 33´´ | H15(2), H16(1), H17(2) |
| 13 | Veracruz, Santa Marta | TUX | 4 | - | 18º 21´ 27´´ | 94° 54´ 39´´ | H16(4) |
| 14 | Oaxaca, Comaltepec | sTMVB | 10 | 1198 | 17° 39´ 16´´ | 96° 20´ 13´´ | H18(7), H19(1), H22(2) |
| 15 | Oaxaca, Chayotepec | sTMVB | 9 | 1137 | 16° 44´ 54´´ | 95° 27´ 24´´ | H19(6), H21(3) |
| 16 | Chiapas, Jitotol | sTMVB | 10 | 1692 | 17° 01´ 47´´ | 92° 50´ 46´´ | H19(1), H23(1), H24(1), H26(2), H27(5) |
| 17 | Chiapas, Coapilla | sTMVB | 13 | 1793 | 17° 09´ 50´´ | 93° 08´ 50´´ | H18(1), H19(4), H25(1), H28(6), H29(1) |
| 18 | Chiapas, Laguna Encantada | sTMVB | 9 | 1422 | 16° 07´ 41´´ | 91° 43´ 49´´ | H19(5), H23(1), H24(2), H28(1) |
| 19 | Chiapas, Angel Albino Corzo | sTMVB | 5 | 1471 | 15° 42´ 02´´ | 92° 43´ 48´´ | H25(5) |
|  |  |  |  |  |  |  |  |

Region abbreviations are as follows: nTMVB = north of the Trans-Mexican Volcanic Belt; sTMVB south of the Trans-Mexican Volcanic Belt; TUX = Sierra de Los Tuxtlas and Sierra de Santa Marta.

**Table S2. Summary statistics of demographic analysis of *Liquidambar styraciflua* grouping samples by (a) populations and (b) groups separated by Trans-Mexican Volcanic Belt (N and S) and TUX region to infer demographic range expansion.**

| Group | *N* | *N_H_* | *h* | | π | | *D*_T_ | *F_S_* | SSD | | Hri |
| --- | --- | --- | --- | --- | --- | --- | --- | --- | --- | --- | --- |
|  |  | | |  | | | | | | | |
| (a) *Populations* | | | | | |  | | | | | |
| 1 | 2 | 1 | - | | - | | - | - | | - | - |
| 2 | 9 | 2 | 0.50±0.12 | | 0.0003±0.000 | | 0.000 | 0.849 | | **0.0219** | **0.2500** |
| 3 | 14 | 3 | 0.53±0.11 | | 0.0004±0.000 | | 0.000 | -0.207 | | **0.0276** | **0.2060** |
| 4 | 11 | 2 | 0.18±0.14 | | 0.0001±0.000 | | 0.000 | -0.409 | | **0.0175** | **0.4380** |
| 5 | 4 | 2 | 0.66±0.20 | | 0.0004±0.000 | | 0.000 | 0.540 | | **0.0898** | **0.5555** |
| 6 | 9 | 3 | 0.41±0.19 | | 0.0003±0.000 | | 0.000 | **-1.081*** | | **0.0086** | **0.1689** |
| 7 | 6 | 4 | 0.86±0.12 | | 0.0019±0.001 | | 0.561 | 0.110 | | **0.0822** | **0.2044** |
| 8 | 10 | 3 | 0.51±0.16 | | 0.0004±0.000 | | 0.000 | -0.271 | | **0.0007** | **0.0819** |
| 9 | 10 | 4 | 0.64±0.15 | | 0.0006±0.000 | | -1.400 | -0.971 | | **0.0013** | **0.0681** |
| 10 | 10 | 1 | - | | - | | - | - | | - | - |
| 11 | 5 | 1 | - | | - | | - | - | | - | - |
| 12 | 10 | 3 | 0.62±0.13 | | 0.0007±0.000 | | 0.000 | 0.602 | | **0.0066** | **0.0518** |
| 13 | 4 | 1 | - | | - | | - | - | | - | - |
| 14 | 10 | 3 | 0.51±0.16 | | 0.0008±0.000 | | 0.819 | 0.800 | | 0.384*** | **0.2913** |
| 15 | 9 | 2 | 0.50±0.12 | | 0.0003±0.000 | | 0.986 | 0.849 | | **0.0219** | **0.2500** |
| 16 | 5 | 1 | - | | - | | - | - | | - | - |

| 17 | 13 | 5 | 0.73±0.09 | 0.0007±0.000 | -1.468 | **-1.260*** | **0.0129** | **0.1464** |
| --- | --- | --- | --- | --- | --- | --- | --- | --- |

| 18 | 9 | 4 | 0.69±0.14 | 0.0007±0.000 | | - | -0.822 | **0.0089** | **-0.0671** |
| --- | --- | --- | --- | --- | --- | --- | --- | --- | --- |
| 19 | 10 | 5 | 0.75±0.12 | 0.0014±0.000 | | 1.463 | -0.552 | 0.4354*** | **0.0627** |
| (b) *Trans-Mexican Volcanic Belt* | | | | |  | | | | |
| N | 90 | 16 | 0.86±0.01 | 0.0019±0.001 | | 0.264 | -3.629 | **0.0222** | **0.0490** |
| S | 56 | 11 | 0.85±0.02 | 0.0012±0.000 | | -0.466 | -3.280 | **0.0028** | **0.0645** |
| TUX | 14 | 3 | 0.48±0.14 | 0.0005±0.000 | | 0.000 | 0.375 | **0.0097** | **0.1208** |

*N* = number of individuals, *N_H_* = number of haplotypes, *h* = gene diversity, π = nucleotide diversity, *D*_T_ = Tajima’s *D*, *F_S_* = Fu’s *F*s, SDD = differences in the sum of squares or mismatch distribution, Hri = Harpending´s raggedness index. n.a. = not available; **P* < 0.05; ***P* < 0.01; ****P* < 0.001. *D*_T_ and *F_S_* positive values are indicative of mutation-drift-equilibrium, which is typical of stable populations, and negative values that result from an excess of rare haplotypes indicate that populations have undergone recent expansions, often preceded by a bottleneck. Significantly negative values (at the 0.05 level) reveal in both tests historic demographic expansion events. Significant (*P* ≤ 0.05) SSD and Hri values indicate deviations from the sudden expansion model. In bold are shown values that are consistent with demographic expansion.

**Table S3.** Summary of coalescent analyses of various models of population structure of *Liquidambar styracifua*.

| Generation time  (years) | Divergence  time (generations) | Effective population size |  | *s*  95% confidence interval | |  |
| --- | --- | --- | --- | --- | --- | --- |
|  |  |  |  | Fragmentation hypothesis | Two-refugia hypothesis | |
|  |  |  |  |  |  | |
| 34 | T1 = 250,000 | 10,000 |  | 32 | 32 | |
|  |  | 50,000 |  | 32–34 | 32–33 | |
|  |  | 100,000 |  | 32–40 | 32–38 | |
|  |  | 1,000,000 |  | 106–127 | 102–120 | |
|  | T2 = 152,941 | 10,000 |  | 32 | 32 | |
|  |  | 50,000 |  | 32–37 | 32–36 | |
|  |  | 100,000 |  | 39–50 | 38–48 | |
|  |  | 1,000,000 |  | 132–151 | 125–144 | |
|  | T3 = 52,941 | 10,000 |  | 32–33 | 32–33 | |
|  |  | 50,000 |  | 48–61 | 32–36 | |
|  |  | 100,000 |  | 72–89 | 38–48 | |
|  |  | 1,000,000 |  | 176–195 | 125–144 | |
|  | T4 = 3,529 | 10,000 |  | 90–108 | 86–104 | |
|  |  | 50,000 |  | 165–185 | 162–177 | |
|  |  | 100,000 |  | 188–206 | 180–200 | |
|  |  | 1,000,000 |  | **217–229** | 171–187 | |
| 124 | T1 = 68,548 | 10,000 |  | 32–33 | 32 | |
|  |  | 50,000 |  | 42–53 | 40–51 | |
|  |  | 100,000 |  | 62–77 | 59–73 | |
|  |  | 1,000,000 |  | 167–188 | 159–179 | |
|  | T2 = 41,935 | 10,000 |  | 32–35 | 32–34 | |
|  |  | 50,000 |  | 55–69 | 52–66 | |
|  |  | 100,000 |  | 82–99 | 78–94 | |
|  |  | 1,000,000 |  | 183–201 | 175–194 | |
|  | T3 = 14,516 | 10,000 |  | 40–51 | 39–51 | |
|  |  | 50,000 |  | 100–118 | 94–112 | |
|  |  | 100,000 |  | 133–155 | 127–146 | |
|  |  | 1,000,000 |  | **206–221** | **200–216** | |
|  | T4 = 967 | 10,000 |  | 153–172 | 144–165 | |
|  |  | 50,000 |  | **201–217** | **195–212** | |
|  |  | 100,000 |  | **210–225** | **207–221** | |
|  |  | 1,000,000 |  | **220–231** | **219–231** | |
|  |  |  |  |  |  | |

Values represent the range of Slatkin and Maddison’s (1989) *s*-parameter from 1000 simulated gene trees, which measures the degree of concordance between the *L. styraciflua* cpDNA gene tree and one of the two models of population structure: fragmentation of populations after isolation in a single refugium and isolation in two refugia. The single refuge-fragmentation model was represented as a tree of 34 localities diverging from a single polytomy and the two-refugia (i.e. vicariance) model represented by two population trees, US and Mesoamerica. The observed *s* value for the cpDNA gene tree was 212. When the observed *s* value falls within the 95% confidence level for any particular model (range in bold), that model is accepted at a maximum *P* = 0.05.
